# HJC0152 suppresses human non–small‐cell lung cancer by inhibiting STAT3 and modulating metabolism

**DOI:** 10.1111/cpr.12777

**Published:** 2020-02-05

**Authors:** Lu Lu, Hang Li, Xin Wu, Jun Rao, Jia Zhou, Saijun Fan, Qiang Shen

**Affiliations:** ^1^ Tianjin Key Laboratory of Radiation Medicine and Molecular Nuclear Medicine Institute of Radiation Medicine Chinese Academy of Medical Sciences and Peking Union Medical College Tianjin China; ^2^ Jiangxi Cancer Hospital Nanchang China; ^3^ Department of Pharmacology and Toxicology The University of Texas Medical Branch Galveston TX USA; ^4^ Department of Genetics Stanley S. Scott Cancer Center Louisiana State University Health Sciences Center Louisiana Cancer Research Center New Orleans LA USA

**Keywords:** HJC0152, lung cancer, metabolism, reactive oxygen species, STAT3

## Abstract

**Objectives:**

Signal transducer and activator of transcription 3 (STAT3) is constitutively activated and overexpressed in many cancers, including non–small‐cell lung cancer (NSCLC). We recently developed HJC0152 as an orally active STAT3 inhibitor. This study focused on investigating HJC0152's effect and mechanism of action in NSCLC.

**Materials and methods:**

We analysed cell proliferation by MTT assays, cell migration by wound healing and transwell assays, protein levels by Western blot, and apoptosis and reactive oxygen species (ROS) level by flow cytometry. A nude mouse tumorigenesis model was established for in vivo experiment. UHPLC‐QTOF/MS was used for untargeted metabolomic relative quantitation analysis.

**Results:**

We found that HJC0152 exhibited activity against human NSCLC cells in vitro and NSCLC xenograft tumours in vivo via regulating STAT3 signalling and metabolism. HJC0152 efficiently reduced NSCLC cell proliferation, promoted ROS generation, induced apoptosis, triggered DNA damage and reduced motility in A549 and H460 NSCLC cells. Moreover, HJC0152 significantly inhibited the growth of A549 xenograft tumours in vivo. HJC0152 also affected metabolism, significantly decreasing and perturbating levels of several metabolites in the purine, glutathione and pyrimidine metabolism pathways.

**Conclusions:**

HJC0152 reduces cellular capacity to scavenge free radicals, leading to ROS generation and accumulation and apoptosis. This study provides a rationale for further developing HJC0152 as a potential therapy for NSCLC and provides insights into the mechanisms by which HJC0152 exerts its anti‐cancer effects.

## 1 INTRODUCTION

Malignant tumours pose a great threat to human health and life and are one of the leading causes of death in the world.^1^ Lung cancer is the second most frequently diagnosed malignant tumour, and the 5‐year survival rate of advanced lung cancer is about 15%.^1^ Worldwide in 2018, lung cancer occurred in 2.1 million people and resulted in 1.76 million deaths.^2^ This makes it the most common cause of cancer‐related death in men and the second most common in women after breast cancer.^2^ Lung cancer can be divided into 2 main pathological types: non–small‐cell lung cancer (NSCLC) and small‐cell lung cancer. NSCLC accounts for 80%‐85% of all lung cancer cases.^3^ In recent years, although some progress has been made in the treatment of NSCLC with surgery, radiotherapy, chemotherapy and targeted therapy, the 5‐year survival rate remains low.^1^ Therefore, there is an urgent need for more effective treatments for NSCLC.

While the aetiology of NSCLC is considered multifactorial, aberrant activation of multiple oncogenes and inactivation of tumour suppressor genes are the primary contributors to abnormal proliferation and reduced apoptosis of pulmonary epithelial cells. Accumulating evidence has shown that signal transducer and activator of transcription 3 (STAT3) is constitutively activated and overexpressed in a variety of malignant tumours, including NSCLC. STAT3 is implicated in cancer cell proliferation and invasion, apoptosis and the cell cycle^4^, ^5^, ^6^ and making it a potential therapeutic target. However, targeting STAT3 with its inhibitors has been a challenge in the past decade due to off‐target effects or poor bioavailability associated with in‐developing STAT3 inhibitors.

Accumulating evidence shows that cellular metabolism plays an important role in the development of cancer; thus, metabolism is a new focus for research on the pathogenesis of cancer and for the search for cancer biomarkers.^7^ Metabolomics is a branch of systems biology similar to genomics, transcriptomics and proteomics. Metabolomic research has contributed important insights to cancer biology and clinical practice in oncology.^8^ Metabolomic studies can use highly sensitive, high‐throughput instrumentation to qualitatively and quantitatively determine changes in the abundance of metabolites during carcinogenesis and tumour development and to screen and identify novel tumour biomarkers.^9^ Recently, metabolomics has been used in research on prostate cancer,^10^ hepatocellular carcinoma,^11^ colorectal cancer,^12^ papillary thyroid carcinoma^13^ and other malignant tumours,^14^, ^15^ leading to an ever‐growing list of metabolites that may be cancer biomarkers or therapeutic targets. Importantly, STAT3 involves in regulating key metabolism pathways in normal and cancer cells.

**Figure 1:**
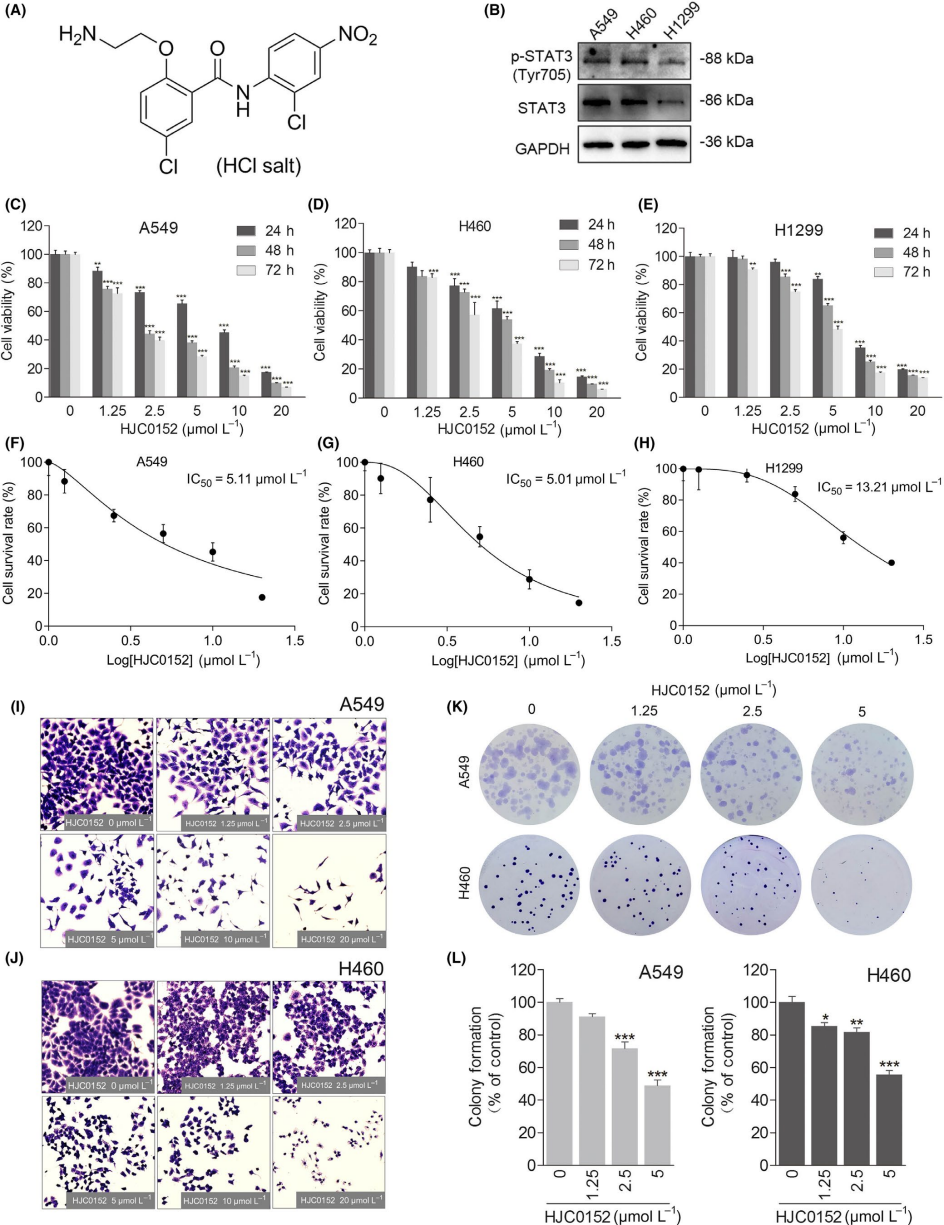
HJC0152 inhibits proliferation of human lung cancer cells. A, Chemical structure of HJC0152. B, Equal amounts of protein from lung cancer cell lines A549, H460, and H1299 were analysed by Western blotting for expression of p‐STAT3 (Tyr705) and STAT3. GAPDH was used as a loading control. C‐E, Results of MTT assays of cell survival. A549 (C), H460 (D), and H1299 (E) cells were treated with the indicated doses of HJC0152 for 24, 48 or 72 h and subjected to MTT assays. Results are presented as the mean ± standard error of 3 independent experiments. F‐H, 24‐h IC_50_ values for HJC0152 in A549 (F), H460 (G), and H1299 (H) cells. I‐J, Representative photographs of cell viability assays (100× magnification). A549 (I) and H460 (J) human NSCLC cells were treated with the indicated concentrations of HJC0152 for 24 h. Cell viability was determined by crystal violet staining. K‐L, Representative photographs of colony formation assays of A549 and H460 cells treated with the indicated concentrations of HJC0152. Results are presented as the mean ± standard error from 3 independent experiments. ^*^ *P* < .05, ^**^ *P* < .01, ^***^ *P* < .001 vs control

We and others recently determined that niclosamide, a Food and Drug Administration‐approved anthelmintic drug,^16^ effectively suppresses the activation, nuclear translocation and transactivation of STAT3.^17^, ^18^ However, the further clinical development of niclosamide for cancer therapy is hindered by its poor water solubility and low oral bioavailability. We previously developed a series of O‐alkylamino‐tethered niclosamide derivatives, including HJC0152 (Figure 1A).^19^ HJC0152 has significantly better (up to 680‐fold) aqueous solubility and oral bioavailability. It also has stronger STAT3‐inhibiting activity than niclosamide.^19^, ^20^

However, little is known about whether HJC0152 has anti‐lung cancer activity, and particularly whether it has effects against NSCLC. In this study, we investigated the anti‐cancer effect of HJC0152 on human NSCLC cell lines in vitro and NSCLC xenograft tumours in vivo. We observed significant inhibition of proliferation and motility in cultured HJC0152‐treated NSCLC cells and inhibition of xenograft tumour growth in mice. Further, STAT3 was found to be involved in NSCLC carcinogenesis and in mediating HJC0152's anti‐NSCLC effects. We then delineated the metabolic alterations that occur in NSCLC cells treated with HJC0152 in this report.

## 2 MATERIALS AND METHODS

### 2.1 Reagents

The design and synthesis of HJC0152 were described in our previous publication.^19^ Antibodies against STAT3 (Cat# 4904), STAT1 (Cat# 9172), STAT5 (Cat# 9363), γ‐H2AX (Cat# 9718) and Alexa Fluor 555 goat anti‐rabbit immunoglobulin G (H + L) (Cat# 4413) were procured from Cell Signaling Technology. Bcl2 (Cat# 12789‐1‐AP) and GAPDH (Cat# HRP‐60004) antibodies were obtained from Proteintech. An antibody against STAT3 phosphorylated at tyrosine residue 705 (p‐STAT3(Tyr705)) (Cat# ab76315) was purchased from Abcam*.* ProLong Gold antifade reagent with 4′,6‐diamidino‐2‐phenylindole (DAPI) (Cat# P36941) was obtained from Thermo Fisher Scientific. All other reagents used were purchased from commercial sources unless otherwise indicated. All reagents were dissolved and used as recommended by their suppliers.

### 2.2 Cell lines and culture conditions

The human NSCLC cell lines A549 and H460 were obtained from ATCC. A549 cells were cultured in Dulbecco's modified Eagle's medium (DMEM)/F12 (HyClone Laboratories) supplemented with 10% foetal bovine serum (FBS) (Biological Industries) and 1% penicillin‐streptomycin solution (Gibco). H460 and H1299 cells were cultured in RPMI‐1640 (HyClone Laboratories) supplemented with 10% foetal bovine serum (FBS) and 1% penicillin‐streptomycin solution. All cells were cultured in a humidified atmosphere (37°C, 5% CO_2_).

### 2.3 Cell proliferation assays with crystal violet staining

Following 24 hours of HJC0152 treatment at different concentrations, cells were fixed in 4% paraformaldehyde in phosphate‐buffered saline (PBS) for 10 minutes. After being washed with PBS, cells were incubated with 0.1% crystal violet solution for 10 minutes. Cells were then gently washed with distilled water and air‐dried.

### 2.4 Cell growth and viability assays

The detection of cell growth and viability was performed with a 3‐(4,5‐dimethylthiazol‐2‐yl)‐2,5‐diphenyltetrazoliumbromide (MTT) assay (5 mg/mL; Sigma). Briefly, A549 or H460 cells, 5 × 10^3^ cells/well, were seeded into 96‐well plates and incubated at 37°C for 24 hours, then exposed to 0, 1.25, 2.5, 5, 10 or 20 μmol/L of HJC0152 for 24, 48 or 72 hours. After treatment, 20 μL of 5 mg/mL MTT was added to each well and incubated for an additional 4 hours. The precipitates of formazan were dissolved in dimethyl sulfoxide, and the absorbance at 490 nm was recorded using a multimode microplate reader (Infinite M200, Tecan). The half‐maximal inhibitory concentration (IC_50_) was calculated using GraphPad Prism 7 software. Each experiment was conducted independently and repeated at least 3 times.

### 2.5 Colony formation assays

A549 or H460 cells were plated in 6‐well plates (800 cells/well) and allowed to attach overnight. The cells were then incubated in the presence or absence of HJC0152 (0, 1.25, 2.5, or 5 μmol/L) at 37°C in 5% CO_2_ for 24 hours. The cell culture medium was replaced every 3 days. After 14 days, cells were washed twice in cold PBS, fixed with methanol and stained with 0.1% crystal violet. Digital images of the plates were obtained as a permanent record of colony counting. Colonies with >50 cells per field were analysed by ImageJ software.

### 2.6 Cell transfection

NSCLC cells were cultured in 6‐well plates for 24 hours and then transfected with small interfering RNAs (siRNAs) (RIBOBIO) using Lipofectamine 2000 reagent (Thermo Fisher Scientific) according to the manufacturer's instructions. The sequences of the STAT3 siRNAs were sense 5′‐3′ CCCGGAAAUUUAACAUUCUTT, antisense 5′‐3′ AGAAUGUUAAAUUUCCGGGTT.

### 2.7 Flow cytometry

To determine the apoptosis rate, cells were treated with HJC0152 (0, 1.25, 2.5 or 5 μmol/L) for 24 hours, washed with PBS and then incubated for 15 minutes in a binding buffer containing Annexin V‐fluorescein isothiocyanate (FITC) and propidium iodide (PI) staining solution (BD Biosciences) before flow cytometric analysis. To determine intracellular reactive oxygen species (ROS) levels, A549 or H460 cells were treated with HJC0152 (0, 1.25, 2.5 or 5 μmol/L) for 24 hours and then preincubated with 10 μmol/L 2,7‐dichlorodihydrofluorescein diacetate (DCFH‐DA) for 30 minutes at 37°C. Images were acquired under a fluorescence microscope, and the mean fluorescence intensity of DCFH‐DA was measured using a flow cytometer (Accuri C6, BD Biosciences) as previously described.^21^

### 2.8 Scratch assays

For the scratch assay, A549 or H460 cells were seeded into 6‐well plates and cultured overnight. The confluent monolayer of cells was scratched with a 10‐μL sterile pipette tip. Suspended cells were washed away with PBS, and medium (with 2% FBS) containing HJC0152 (0, 0.625, 1.25 or 2.5 μmol/L) was added to the wells. The plates were maintained at 37°C for 48 hours. At 0, 24 and 48 hours after scratching, representative images of the gaps from several randomly selected fields of the plates were photographed with an inverted light microscope. All experiments were repeated 3 times under each condition.

### 2.9 Transwell migration/invasion assays

Migration and invasion assays were performed using 24‐well insert Transwell permeable supports containing a polycarbonate filter with an 8‐μm pore size (Corning, Inc). For migration assays, HJC0152‐treated A549 (5 × 10^5^ cells/mL) or H460 (1 × 10^6^ cells/mL) cells were suspended in 100 µL DMEM containing 0.1% bovine serum albumin (BSA) and placed in the upper compartment of the chamber. For the invasion assays, A549 (1 × 10^6^ cells/mL) or H460 (2 × 10^6^ cells/mL) cells were suspended in 300 µL DMEM containing 0.1% BSA and were added onto the invasion assay instrument ECM550 (Millipore). Medium with 10% FBS was added into the bottom chambers of the wells (500 µL/well). All cells were incubated at 37°C in an atmosphere of 5% CO_2_. After 16 hours, cells that had not penetrated the membrane and those on the bottom of the upper chamber were removed with cotton swabs. The remaining cells, those that had migrated through the pores, were fixed with 4% paraformaldehyde in PBS for 20 minutes and stained with 0.1% crystal violet solution for 15 minutes. Cellular migration and invasion were evaluated by counting the number of cells penetrating through the membrane onto the lower surface, which was performed using a light microscope (Olympus, CX31) at 100× magnification. Five fields were randomly selected for analysis.

### 2.10 Protein extraction and Western blotting

After treatment, cells were prepared in radioimmunoprecipitation assay buffer supplemented with protease and phosphatase inhibitors (Roche) at 4°C for 30 minutes. The lysates were then centrifuged (13 000 × *g*, 4°C) for 15 minutes to obtain total protein lysates. Protein expression was analysed using Western blotting as described previously.^17^, ^20^, ^21^ The expression level of GAPDH was used as the loading control.

### 2.11 Immunofluorescence microscopy

A total of 2 × 10^4^ cells was grown with cell culture medium and treated with designated concentrations of HJC0152 on chambered slides. At designated times after treatment, cells were fixed with 4% paraformaldehyde in PBS and then permeabilized with 0.1% Triton X‐100 (Solarbio) in PBS for 20 minutes at room temperature. Cells were subsequently washed and blocked with 5% BSA (Amresco) in PBS for 60 minutes at room temperature. Primary antibodies against γ‐H2AX were applied to the cells and incubated at 4°C overnight. After being washed with PBS 3 times, the cells were incubated with fluorescence‐conjugated secondary antibodies for 60 minutes at room temperature and then washed 3 times with PBS. Cell nuclei were counterstained with DAPI. Cells were visualized using a fluorescent microscope (Thermo Fisher) or confocal microscope.

### 2.12 Immunohistochemical staining

Details of the immunohistochemical analyses were described previously.^20^ Briefly, tumour tissues were harvested, fixed in 4% paraformaldehyde/PBS, dehydrated, embedded in paraffin and cut into 4‐μm‐thick sections. Deparaffinized sections were rehydrated and stained using specific antibodies against p‐STAT3 (Tyr705), Ki‐67, or cleaved caspase‐3, then stained with biotinylated secondary antibodies. Detection was performed with an avidin‐biotin horseradish peroxidase complex and 3,3′‐diaminobenzidine (BD Biosciences) as the chromogen. All slides were analysed, and representative photographs were taken using an Olympus CX31 microscope (Olympus America) at 400× magnification.

### 2.13 Animals and xenograft tumour model

All animal experiments were carried out in accordance with the Guide for the Care and Use of Laboratory Animals of the Institutional Animal Care and Use Committee of IRM, CAMS (Permit Number 1526, April 7, 2015). BALB/c athymic (nu/nu) mice (male, 6‐week‐old and body weight approximately 18 g) were purchased from Beijing HFK Bioscience Co., Ltd and housed in the certified animal facility (specific pathogen‐free level, individually ventilated cages, independent ventilation system) at the Institute of Radiation Medicine (IRM), Chinese Academy of Medical Sciences (CAMS). A total of 5 × 10^6^ A549 cells were subcutaneously injected into the flank of mice to establish xenograft tumours. Tumours were allowed to grow to an average volume of 100 mm^3^ before initiation of therapy, as described previously.^17^, ^22^ Tumour‐bearing mice were randomly divided into 2 treatment groups (5 mice per group): vehicle control (dimethyl sulfoxide) or HJC0152 (7.5 mg/kg). The schedules for the administration of HJC0152 are provided in the figure legends. Tumour size was measured every three days using a vernier calliper, and the tumour volume was calculated from two‐dimensional measurements (mm) using the following formula: Tumour volume = length × width^2^/2. The tumour volume doubling time (TVDT) was calculated using the following formula: TVDT = [log2 × t]/[log (V_F_/V_1_)], where V_F_ is the final tumour volume, V_1_ is the initial treatment tumour volume, and T is the time interval between the first treatment and final day. Mice were euthanized by cervical dislocation while under anaesthesia on the 30th day after tumour cell inoculation, and the tumours were excised for further pathological examination.

### 2.14 Untargeted metabolomic relative‐quantitative analyses

The cell samples were collected in 15‐mL Vacutainer tubes and then centrifuged for 15 minutes (1500 × *g*, 4°C). Aliquots of the samples were stored at −80°C for use in ultra‐high‐performance liquid chromatography equipped with quadrupole time‐of‐flight mass spectrometry (UHPLC‐QTOF/MS). The samples were thawed at 4°C, and 100‐μL aliquots were mixed with 400 μL of cold methanol/acetonitrile (1:1, v/v) to remove the protein. The mixture was centrifuged for 20 minutes (14 000 × *g*, 4°C). The supernatants were dried in a vacuum centrifuge. Metabolic profiling of cell samples was performed using an ultra‐high‐performance liquid chromatography (1290 Infinity LC, Agilent Technologies) coupled with a quadrupole time‐of‐flight system (AB Sciex TripleTOF 6600, AB SCIEX) at Shanghai Applied Protein Technology Co., Ltd. Detailed experimental procedures for mass spectrometry are provided in the Appendix S1. The raw mass spectrometry data (wiff.scan files) were converted to MzXML files using ProteoWizard MSConvert before being imported into freely available XCMS software. For peak picking, the following parameters were used: centWave m/z = 25 ppm, peakwidth = c (10, 60), prefilter = c (10, 100). For peak grouping, the parameters bw = 5, mzwid = 0.025, minfrac = 0.5 were used. In the extracted ion features, only variables having more than 50% of the nonzero measurement values in at least 1 group were kept. Metabolites were identified by comparing their mass spectra with an in‐house database established using available authentic standards.

After normalization to the total peak intensity, the processed data were uploaded and imported into SIMCA‐P (version 14.1, Umetrics) for multivariate analysis, including Pareto‐scaled principal component analysis (PCA) and orthogonal partial least‐squares discriminant analysis (OPLS‐DA). Sevenfold cross‐validation and response permutation testing was used to evaluate the robustness of the model. The variable importance in the projection (VIP) value of each variable in the OPLS‐DA model was calculated to indicate its contribution to the classification. Metabolites with a VIP value >1 were entered into a univariate Student *t* test to determine the significance of their alterations in the treated samples. *P* values <.05 were considered statistically significant.

The methods used for the bioinformatic analysis of metabolism are described in the Appendix S1.

### 2.15 Statistical analysis

All experiments were performed with at least 3 independent replicates. All data are presented as mean ± standard error of the mean. Statistical comparisons between 2 groups were made using the Student *t* test. Differences between experimental groups were assessed for statistical significance using a 1‐way analysis of variance with repeated measures followed by *post hoc* comparisons using Tukey's multiple paired comparison test. Differences were considered statistically significant at *P* < .05. All analyses were performed using GraphPad Prism software.

## 3 RESULTS

### 3.1 HJC0152 inhibits the proliferation of human lung cancer cells

We initially determined p‐STAT3 (Tyr705) level in non‐squamous NSCLC cells, A549, H460, and H1299. All tested lines had constitutively activated p‐STAT3 (Tyr705) (Figure 1B). Particularly, strong activation of STAT3 was evident in the A549 and H460 cells. Next, we characterize the effect of HJC0152 on NSCLC cell proliferation, and A549, H460 and H1299 cells were treated with HJC0152 in vitro for 24, 48 or 72 hours, and cell viability was determined by MTT assays. As shown in Figure 1C‐E, HJC0152 showed a significant inhibitory effect that was both concentration‐ and time‐dependent. The IC_50_ values of HJC0152 against A549, H460 and H1299 cells were 5.11, 5.01 and 13.21 μmol/L, respectively (Figure 1F‐H). These findings suggest that HJC0152 has a strong cytostatic and cytotoxic effect on NSCLC cells and that NSCLC cells with higher p‐STAT3 (Tyr705) levels are more sensitive to HJC0152.

To further evaluated the anti‐proliferation activity of HJC0152 in 2 human NSCLC cell lines that with high level of p‐STAT3 (Tyr705), A549 and H460 cells were treated with different concentrations of HJC0152 for 24 hours, and significant morphological changes and cell death were observed (Figure 1I and J). In addition, HJC0152‐treated NSCLC cells formed significantly fewer colonies and significantly smaller colonies than did untreated control cells (Figure 1K and L). Collectively, these results demonstrate that HJC0152 has a strong inhibitory effect on the growth of NSCLC cells.

### 3.2 STAT3 activation is positively correlated with NSCLC, and HJC0152 inhibits constitutive STAT3 activation in NSCLC cells

**Figure 2:**
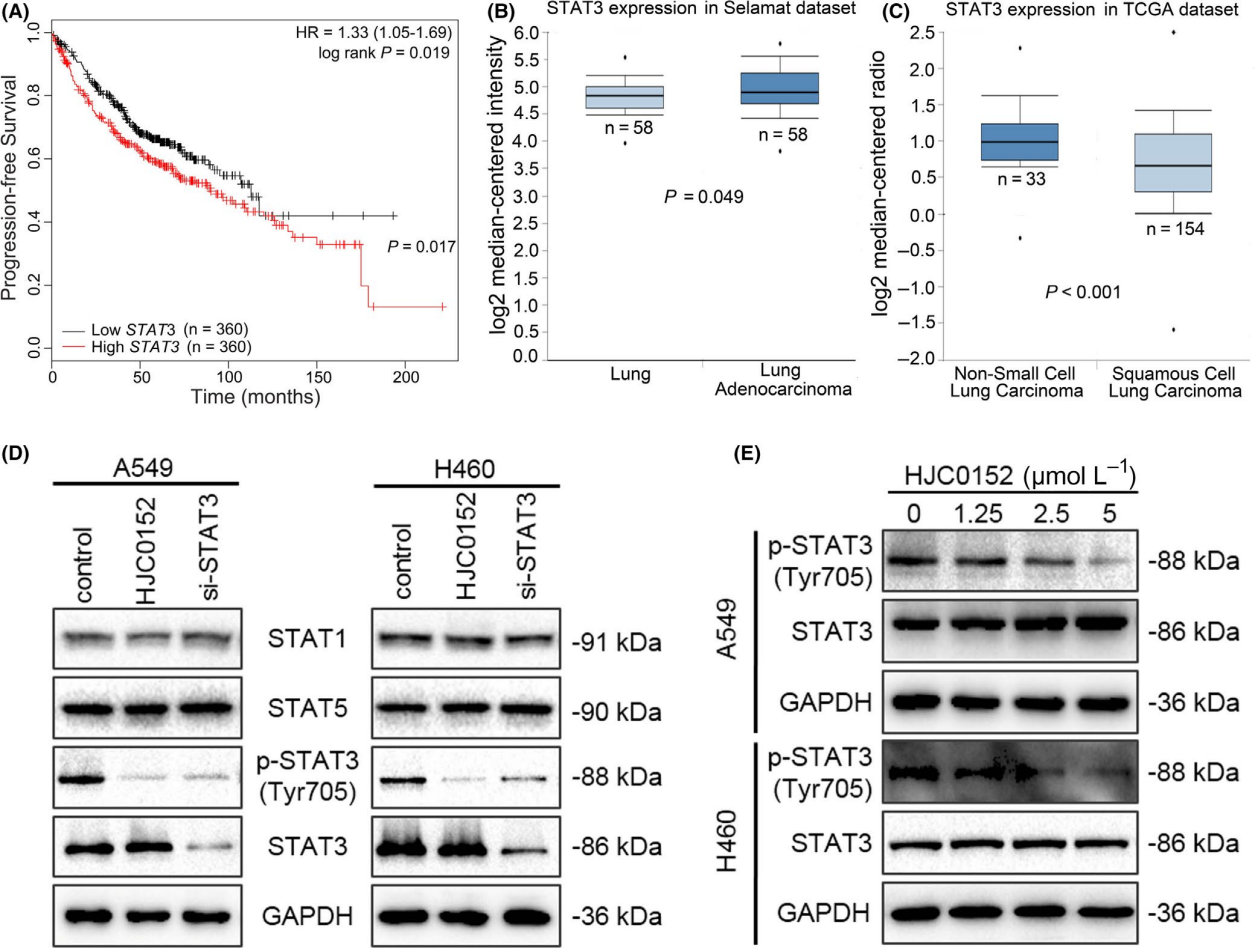
Role of STAT3 in NSCLC. A, Kaplan‐Meier curves showing progression‐free survival in patients with lung adenocarcinoma with high and low STAT3 mRNA expression. B, Box plot comparing STAT3 mRNA levels in normal lung tissue and lung adenocarcinoma tissue. Data were retrieved from the Oncomine database. C, Box plot comparing STAT3 mRNA levels in NSCLC and lung squamous cell carcinoma tissues. Data were retrieved from Oncomine. D, Representative Western blots showing effects on protein expression of STAT3 siRNA and of HJC0152 treatment. GAPDH was used as a loading control. E, Western blots showing that HJC0152 treatment decreased p‐STAT3 (Tyr705) expression in both NSCLC cell lines. GAPDH was used as a loading control

We assessed the prognostic value of STAT3 expression in clinical samples using the Kaplan‐Meier plotter platform (http://www.kmplot.com).^23^ The samples were grouped according to the median (or upper or lower quartile) expression of STAT3 gene, and then, the two groups were compared by a Kaplan‐Meier plot. The patients in the cohorts were filtered using the term “adenocarcinoma” before running the analysis. The results showed that high levels of STAT3 expression were associated with shorter progression‐free survival durations in patients with lung adenocarcinoma (Figure 2A). Data from the Oncomine database^24^ confirmed that STAT3 was more highly expressed in lung adenocarcinoma tissues than in corresponding normal lung tissue and was also expressed significantly more highly in NSCLC than in squamous cell lung carcinoma (Figure 2B and C).

Next, we determined the expression level of STAT3 and 2 other key members of the STAT family of transcription factors, STAT1 and STAT5. A STAT3 siRNA was used to block STAT3 expression, and the levels of the 3 STAT proteins were determined. As shown in Figure 2D, STAT3 siRNA inhibited the expression of STAT3 and p‐STAT3 (Tyr705). Lower p‐STAT3 (Tyr705) protein levels were found in HJC0152‐treated A549 and H460 cells than in control cells, whereas no difference in the levels of STAT1 and STAT5 was found (Figure 2D). Furthermore, HJC0152 treatment inhibited p‐STAT3 (Tyr705) expression in NSCLC cells in a dose‐dependent manner (Figure 2E).

### 3.3 HJC0152 induces ROS accumulation and apoptosis in human NSCLC cells

**Figure 3:**
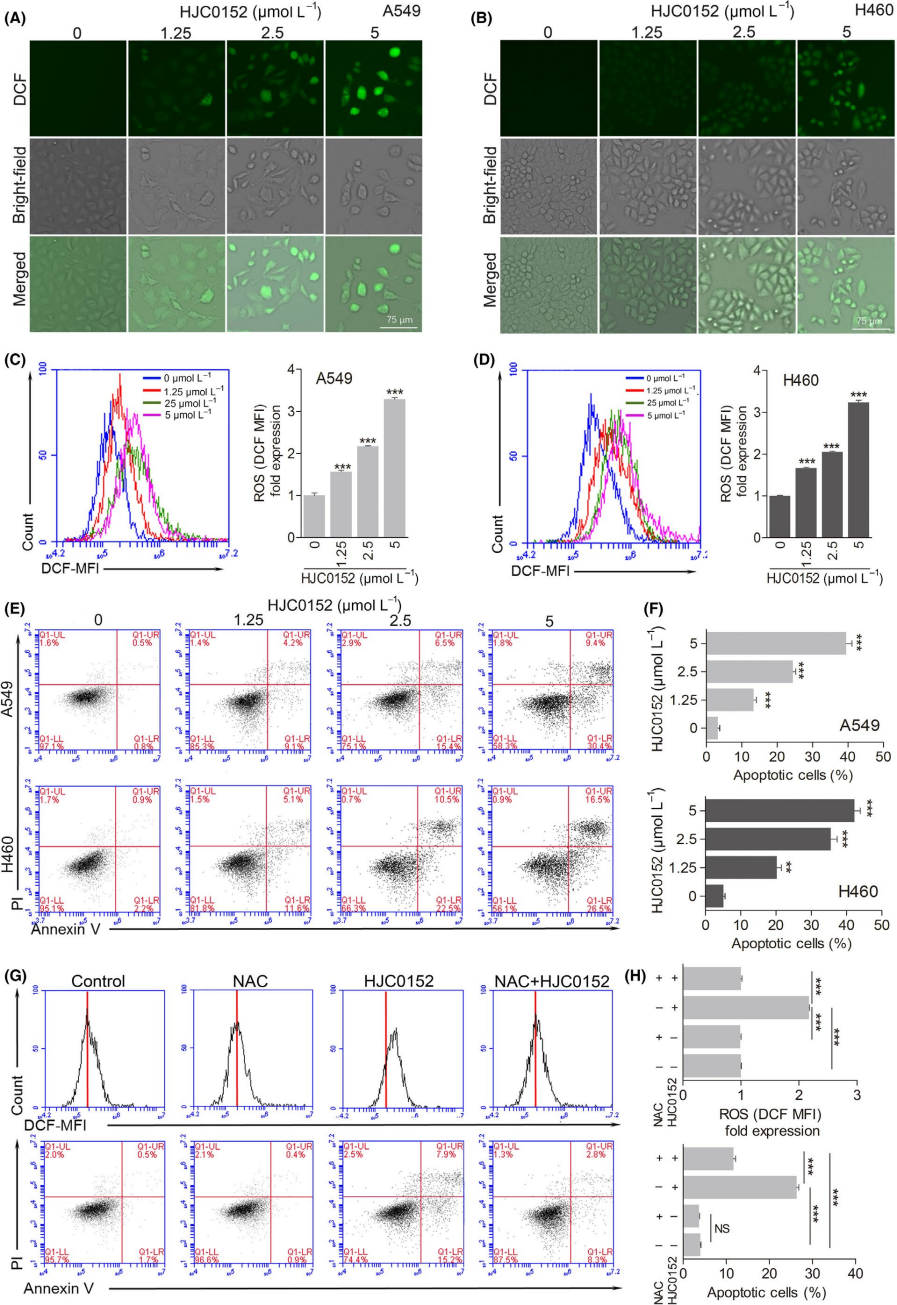
HJC0152 induces reactive oxygen species (ROS) accumulation and apoptosis in human lung cancer cells. A‐D, Intracellular ROS generation induced by treatment with HJC0152. (A and B) Representative fluorescence microscopy images (100× magnification) of DCFH‐DA staining in A549 and H460 cells treated with the indicated concentrations of HJC0152 for 24 h. Scale bar, 75 μm. (C and D) Flow cytometry results showing ROS levels in A549 and H460 cells treated with the indicated concentrations of HJC0152 for 24 h. Data are representative of 3 independent experiments. ^***^ *P* < .001 vs control. E‐F, Flow cytometry analysis showing rates of apoptosis in A549 and H460 cells treated with HJC0152. E, Representative fluorescence‐activated cell sorting profiles of A549 and H460 cells treated with the indicated concentrations of HJC0152 for 24 h. F, Quantification of Annexin V–positive and propidium iodide–positive cells following treatment with the indicated concentrations of HJC0152. ^**^ *P* < .01, ^***^ *P* < .001 vs control. G‐H, HJC0152‐induced ROS elevation partially contributes to the apoptosis of A549 cells. The A549 cells were pre‐treated with NAC (10 mmol/L) for 1 h before 2.5 μmol/L HJC0152 treatment, then stained with DCF‐DA and Annexin V/PI, respectively. G, Flow cytometry analysis showing ROS levels and rates of apoptosis in A549. H, Quantification of ROS levels and rates of apoptosis in A549 cell. ^***^ *P* < .001 vs control

Reactive oxygen species are normal products of cell metabolism,^25^ but they also play an important role in tumorigenesis and cancer progression.^26^ Therefore, to gain further insight into the mechanism of HJC0152‐induced cytotoxicity in NSCLC cells, we evaluated intracellular ROS levels using the fluorescent probe DCFH‐DA. As the concentration of HJC0152 increased, the fluorescence intensity observed by fluorescence microscopy in A549 and H460 cells gradually increased over that of control cells (Figure 3A and B). Flow cytometry also indicated a significant dose‐dependent increase of intracellular ROS generation in treated A549 and H460 cells (Figure 3C and D). These results indicate that HJC0152 induces ROS accumulation in NSCLC cell lines.

**Figure 4:**
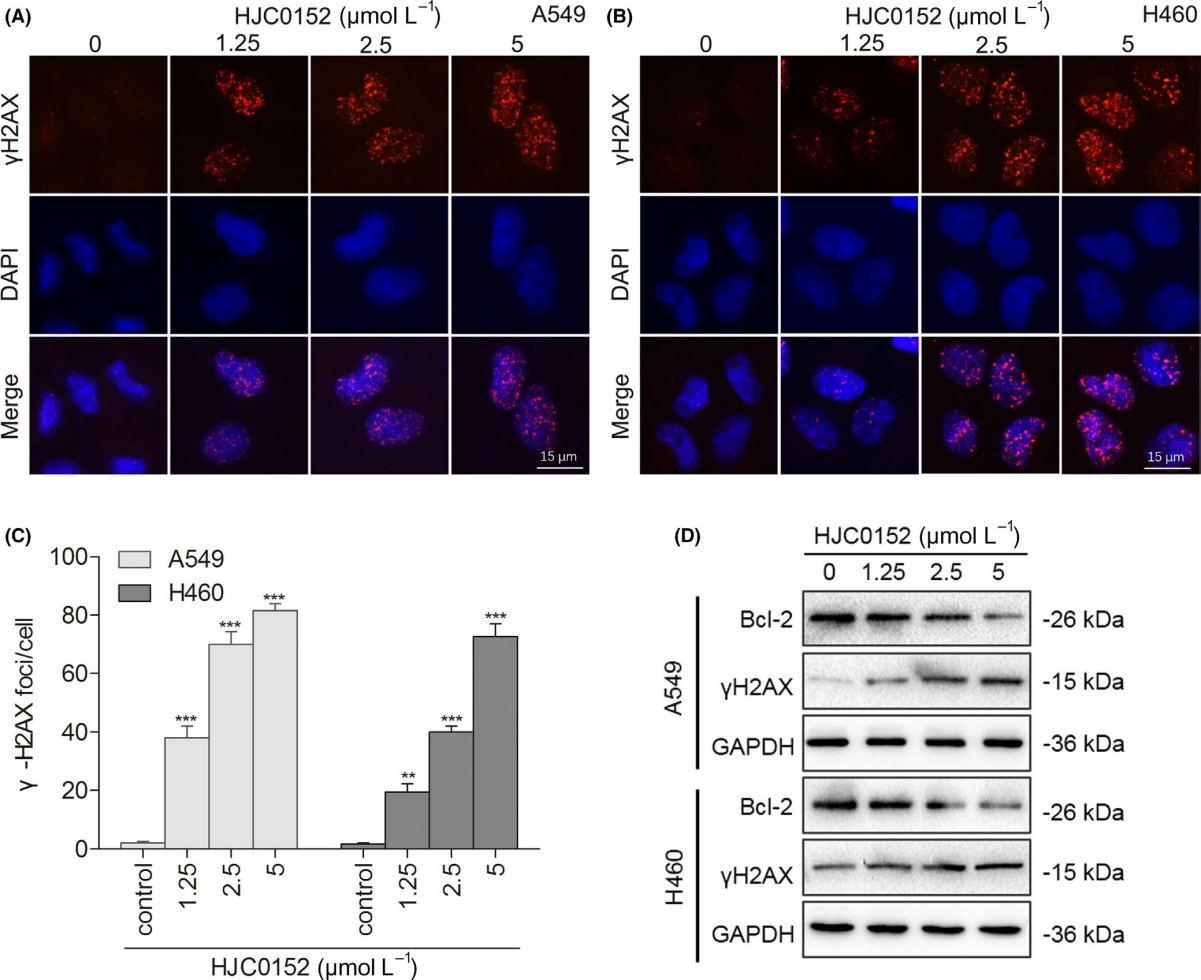
HJC0152 induces DNA damage in human lung cancer cells. A‐B, Representative immunofluorescence images of nuclei (blue), γ‐H2AX foci (red) and complexes (400× magnification) of A549 and H460 NSCLC cells treated with the indicated concentrations of HJC0152. Scale bar, 15 μm. C, Quantification of γ‐H2AX foci per nucleus. Data are mean ± standard error from 3 independent experiments. ^**^ *P* < .01, ^***^ *P* < .001 vs control. D, Western blots showing that HJC0152 treatment at the indicated concentrations decreased the protein levels of Bcl‐2 and γ‐H2AX. GAPDH was used as a loading control

Previously, we found that oxidative stress is an early upstream event of apoptosis in breast cancer cells.^17^ To assess whether HJC0152 induces apoptosis in lung cancer cells, we performed annexin V‐FITC/PI double staining and analysis using flow cytometry.^17^ The results showed that treatment with increasing concentrations of HJC0152 induced a corresponding increase in the percentage of apoptotic cells (Figure 3E and F). This finding, that HJC0152‐induced apoptosis in A549 and H460 cells, was supported by Western blotting. Protein levels of Bcl‐2, a marker of apoptotic cells, decreased in a dose‐dependent manner after HJC0152 treatment (Figure 4D). Taken together, these findings showed that HJC0152 induces ROS accumulation and apoptosis in NSCLC cells.

In addition, pre‐treatment with antioxidants N‐acetyl‐L‐cysteine (NAC), a ROS scavenger,^27^ completed blocked ROS induction by HJC0152 (Figure 3G and H), and partially protected cells from HJC0152‐induced apoptosis (Figure 3G and H). These results suggest that quenching ROS by NAC partly abrogated HJC0152‐induced apoptosis. Therefore, HJC0152‐induced ROS partially contributes to apoptosis of A549 cells.

### 3.4 HJC0152 triggers DNA damage in human lung cancer cells

Many anti‐cancer drugs work by inducing DNA damage in rapidly dividing cancer cells.^28^ Phosphorylated H2AX (γ‐H2AX), a phosphorylated variant of histone 2A, is a well‐known biomarker of DNA double‐strand breaks, which initiate the DNA damage response in mammalian cells.^29^ With DNA damage, formation of γ‐H2AX foci increases in the nucleus. To determine whether HJC0152 triggers a DNA damage response in NSCLC cells, we evaluated γ‐H2AX foci by using immunofluorescence. HJC0152 treatment significantly increased γ‐H2AX foci formation over levels observed in control cells (Figure 4A‐C). These results were further supported by Western blotting analysis showing that the protein level of γ‐H2AX increased in a dose‐dependent pattern (Figure 4D). Together, these data showed that HJC0152 induces DNA damage in NSCLC cells.

### 3.5 HJC0152 suppresses the migration and invasion of NSCLC cells

**Figure 5:**
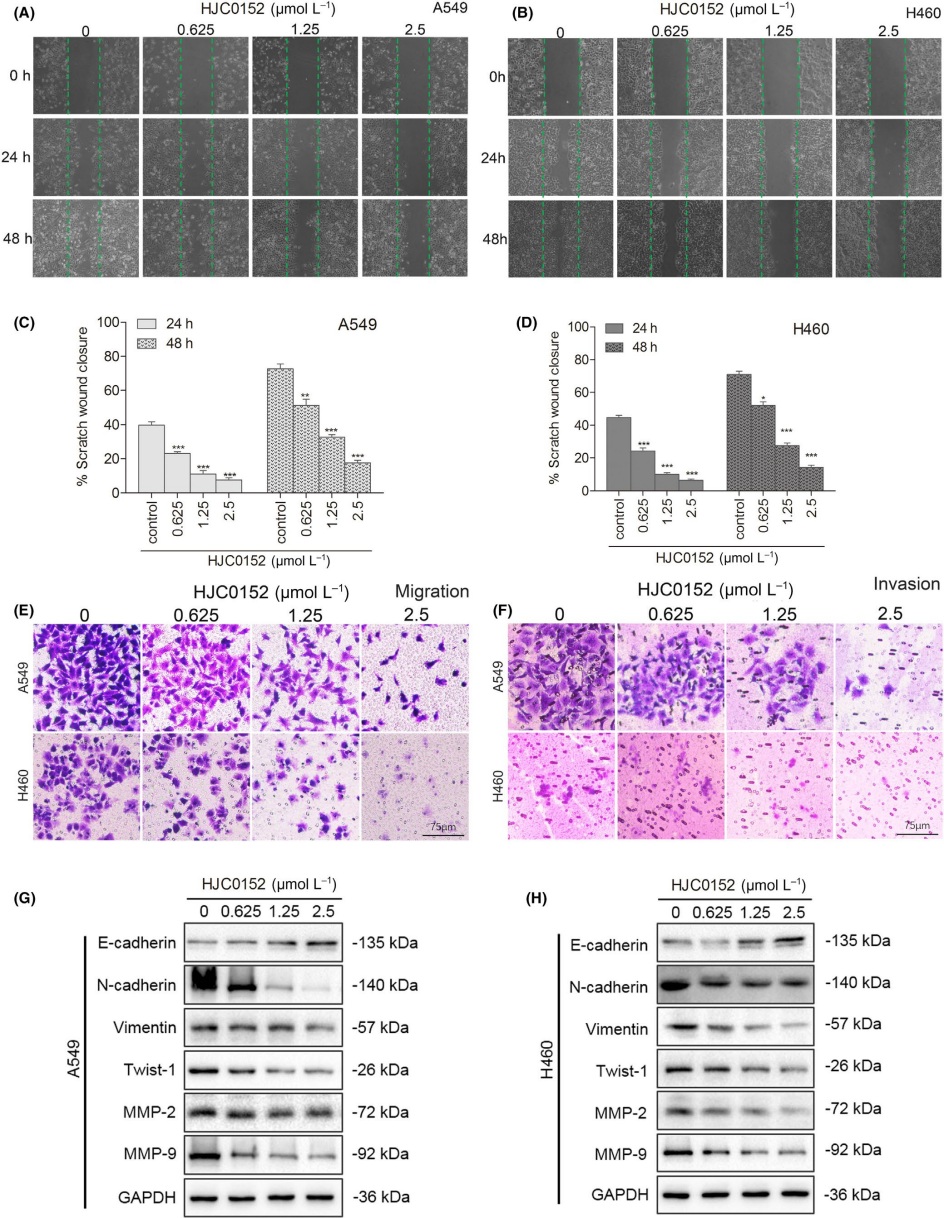
HJC0152 suppresses migration and invasion of NSCLC cell lines. A‐B, Representative images of scratch assays showing wound healing (100× magnification). C‐D, Quantification of the percentage of scratch wound closure at 24 and 48 h. Data are presented as mean ± standard error from 3 independent experiments. ^*^ *P* < .05, ^**^ *P* < .01, ^***^ *P* < .001 vs control. E‐F, Representative images of Transwell migration and invasion assays of A549 and H460 cells treated with the indicated concentrations of HJC0152 (100× magnification). Scale bar, 75 μm. G‐H, Western blots showing effect of treatment with the indicated concentrations of HJC0152 on the protein levels of matrix metalloproteinases and EMT‐associated biomarkers. GAPDH was used as a loading control

We next measured the effect of HJC0152 on the motility of NSCLC cell lines. In scratch assays, HJC0152 treatment delayed wound healing in A549 and H460 cell lines (Figure 5A‐D). Consistent with the results of the scratch assays, HJC0152 inhibited NSCLC cell migration and invasion in a dose‐dependent manner in Transwell assays (Figure 5E‐F, and Figure S1). These results suggest that HJC0152 suppresses the migration and invasion ability of NSCLC cells.

The matrix metalloproteinase (MMP) family plays a key role in facilitating tumour metastasis. In particular, reduction of the secretion and activity of MMP‐2 and MMP‐9 has been shown to inhibit cancer cell motility and metastasis.^30^ In both A549 and H460 cell lines, HJC0152 inhibited the protein expression of MMP2 and MMP9, suggesting that HJC0152 attenuates the ability of these MMPs to degrade the extracellular matrix (Figure 5G and H). Epithelial‐mesenchymal transition (EMT), a biological process in which epithelial cells transform into mesenchymal cells, is regarded as one of the most important mechanisms of cell migration and tumour metastasis, which is closely related to clinical outcomes.^31^, ^32^ EMT is characterized by the loss of expression of proteins that promote cell‐to‐cell contact, such as E‐cadherin, and gain of mesenchymal markers, such as N‐cadherin and vimentin. Therefore, we determined whether the expression of these EMT‐related proteins was influenced by HJC0152 treatment. HJC0152 increased the expression of E‐cadherin but decreased the expression of N‐cadherin and vimentin (Figure 5G and H), suggesting that HJC0152 inhibits acquisition of mesenchymal characteristics in NSCLC cells. Taken together, these results demonstrate that HJC0152 inhibits migration and invasion of NSCLC cells by regulating the EMT process.

### 3.6 HJC0152 significantly suppresses the growth of NSCLC xenograft tumours in vivo

**Figure 6:**
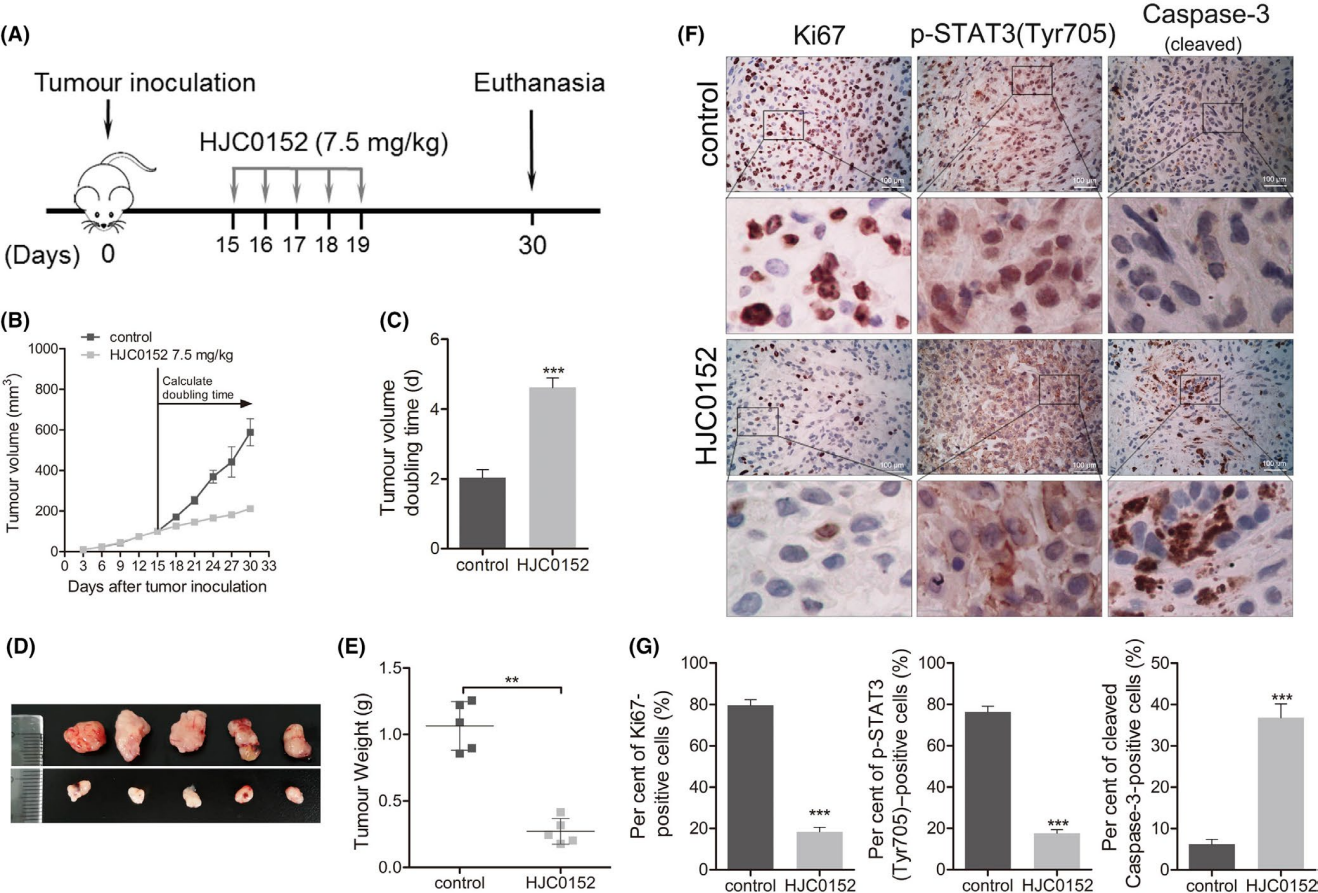
HJC0152 exhibits antitumour activity in an in vivo model of lung cancer. A, Mice bearing NSCLC xenograft tumours developed from A549 cells were treated with vehicle (control) or HJC0152 (7.5 mg/kg/d), as illustrated in the diagram. B, Tumour growth curves of groups as indicated. C, Tumour volume doubling time (time for a tumour to double in volume) of groups. Data are presented as mean ± standard error. ^***^ *P* < .001 vs control. D, Images of dissected tumours from nude mice (n = 5). E, Weight of resected xenograft tumours from nude mice. Data are presented as mean ± standard error. ^**^ *P* < .01 vs control. F, Representative images (400× magnification) of A549 xenografts stained for Ki‐67, p‐STAT3 (Tyr705) and cleaved caspase‐3. G, Quantification of Ki‐67, p‐STAT3 (Tyr705) and cleaved caspase‐3. ^***^ *P* < .001 vs control

We previously reported that HJC0152 has anti‐cancer effects in animal models of human breast cancer,^19^ head and neck squamous cell carcinoma,^20^ glioma,^33^ and gastric cancer.^34^ To establish the in vivo anti‐cancer efficacy of HJC0152 against human lung cancer, we generated a mouse xenograft lung cancer model by subcutaneously injecting A549 NSCLC cells into mice (Figure 6A). HJC0152 given to A549‐bearing mice significantly retarded the tumour growth rate compared to that of the control group. Tumour growth appeared slow, as indicated by the mean tumour volume doubling time (TVDT), which was 2.03 and 4.62 days in the control and HJC0152 groups, respectively (Figure 6B and C). The tumour weight in mice treated with HJC0152 was significantly lower than that of control mice (Figure 6D and E). These results demonstrate that HJC0152 has significant antitumour activity in a NSCLC xenograft model raised from A549 cells. Immunohistochemical assays showed decreased intensity of Ki67, p‐STAT3 (Tyr705), and cleaved caspase‐3 staining in tumours from mice treated with HJC0152 (Figure 6F and G). Collectively, these results suggest that HJC0152 significantly suppressed the in vivo growth of human lung cancer xenografts in mice.

### 3.7 HJC0152 reprograms metabolism of human NSCLC cells

**Figure 7:**
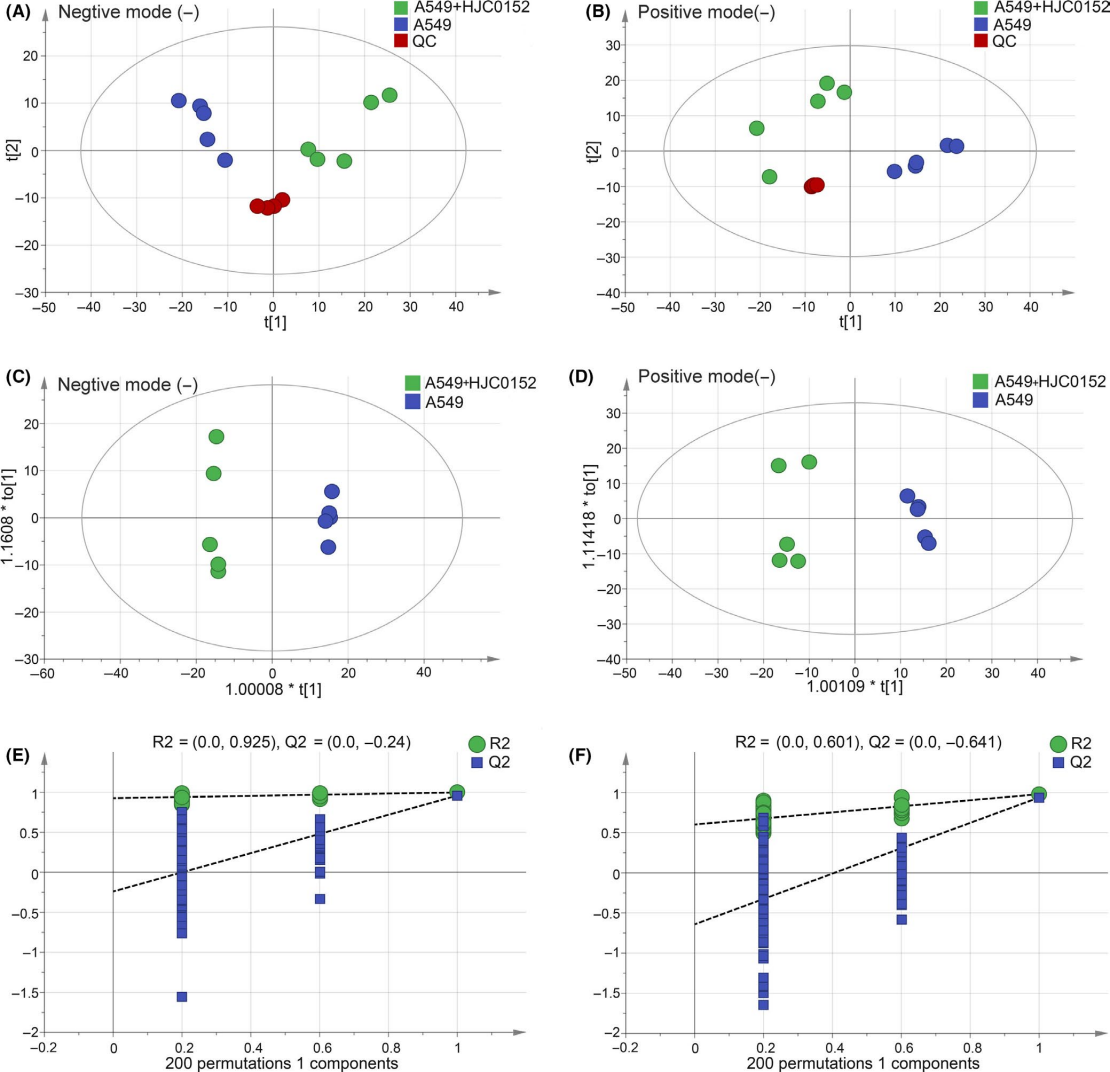
Metabolomic analysis of A549 NSCLC cells treated with HJC0152. A‐B, The principal component analysis (PCA) score plots of the quality control (QC) samples and NSCLC samples. All QC samples were clustered in the PCA plots, and the error was within 2 standard deviations. A, negative ion, B, positive ion. C‐D, Orthotopic partial least‐squares discriminant analysis (OPLS‐DA) score plots. C, negative ion, D, positive ion. E‐F, Permutation tests for the OPLS‐DA score plots E, negative ion, F, positive ion

**Figure 8:**
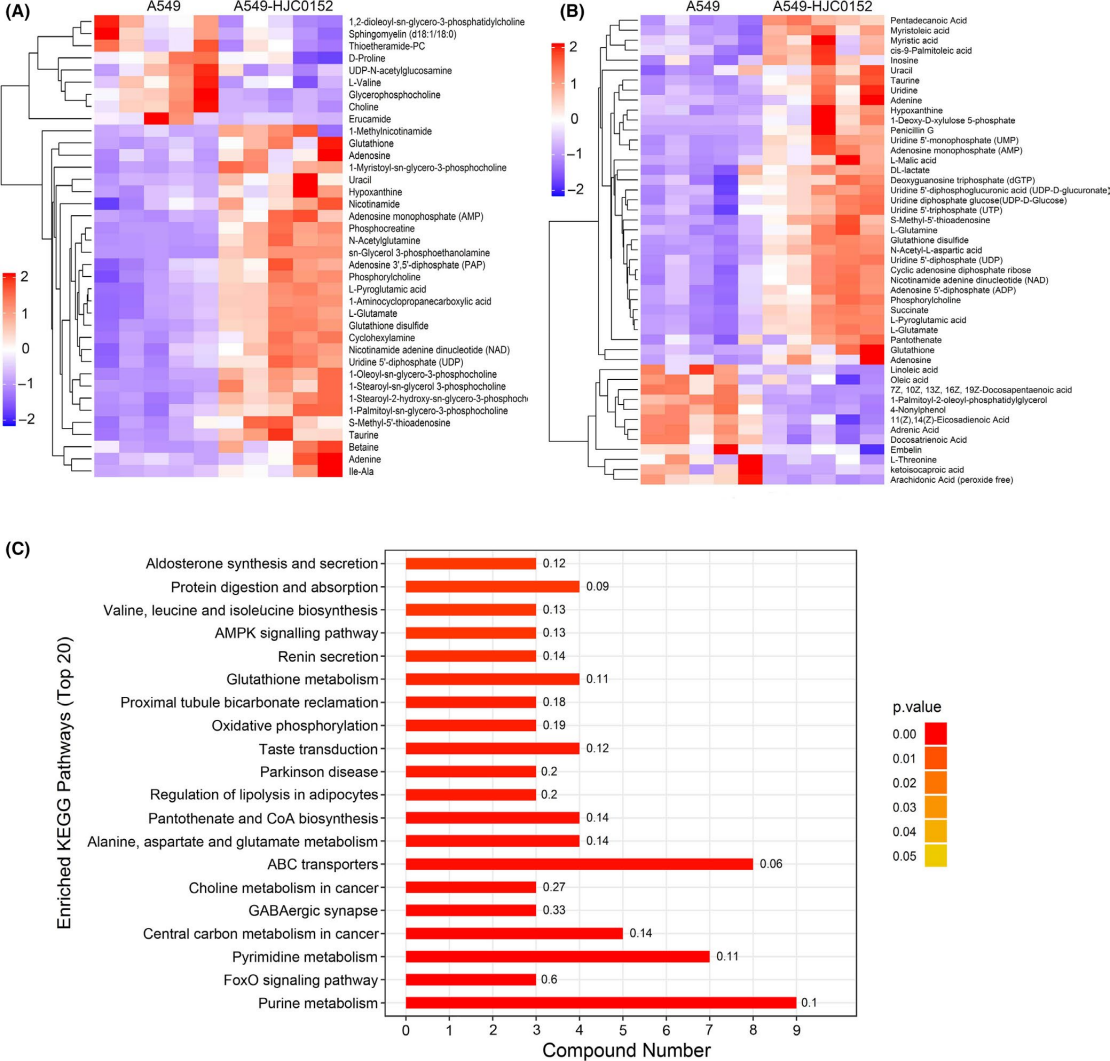
Altered metabolites and metabolic pathways in HJC0152‐treated A549 NSCLC cells. A‐B, Heat plot of the differentially expressed metabolites in HJC0152‐treated and untreated A549 lung cancer cells. A, negative ion, B, positive ion. C, Enriched KEGG pathways in HJC0152‐treated A549 cells

Our previous studies and a report from other groups have shown that STAT3 is a primary target of HJC0152 in breast cancer, head and neck squamous cell carcinoma, glioma, and gastric cancer.^19^, ^20^, ^33^, ^34^ In this study, we showed that STAT3 expression is a prognostic factor for NSCLC patients (Figure 2A‐C), and that STAT3 is activated in NSCLC cells (Figure 2D and E). We hypothesized that HJC0152 alters cellular metabolism by targeting STAT3 and its signalling during lung carcinogenesis. We therefore characterized the metabolic alterations in A549 NSCLC cells treated with HJC0152 using UHPLC‐QTOF/MS technology. In total, 5160 positive‐ion‐mode and 5227 negative‐ion‐mode features were identified in 10 samples from A549 cells treated with or without HJC0152. As shown in Figure 7A and B, quality control samples in both negative and positive ion modes were clustered together in the PCA score plot, indicating satisfactory stability and reproducibility of the analysis platform. Moreover, the total ion chromatograms showed no drifting retention time during the whole run sequence (Figure S2A‐D), indicating that the UHPLC‐QTOF/MS results were qualified for statistical analyses. Notably, a clear separation between samples from treated and untreated A549 cell samples was observed in PCA analysis under both ionization modes, indicating marked metabolic differences between control samples and those treated with HJC0152 (Figure 7A and B). To further profile differential metabolism in A549 cells with or without HJC0152 treatment, we next performed OPLS‐DA analysis in both negative and positive modes, and permutation tests with 200 iterations to prevent the overfitting of the OPLS‐DA model (Figure 7C‐F). According to multivariate and univariate statistical significance criteria (VIP ≥ 1 and *P* ≤ .05), 47 metabolites were differentially expressed in control and HJC0152‐treated A549 cells in the negative mode, and 38 differentially expressed metabolites were identified in the positive mode (Figure 8A and B). HJC0152 was shown to reduce the levels of several highly abundant metabolites and increased the levels of low‐abundance metabolites. Of all the significantly altered metabolites, under an indistinguishable ion mode, 14 were increased and 48 were decreased, with an amplitude of variation ranging from 0.02‐fold to 7.69‐fold. Detailed information on these metabolites is shown in Table 1.

**Table 1:** Identified differentially expressed metabolites between HJC0152‐treated and control A549 cells

Metabolite	Rt (s)	m/z	ESI^±^	VIP	FC	*P*‐value
Adenosine monophosphate (AMP)	346.05	406.48	±	1.66	0.36	.002
Adenosine	268.1	162.65	±	3.87	0.29	.019
Adenine	134.05	155.07	±	2.6	0.39	.033
Inosine	267.07	210.71	−	1.41	0.51	.01
Hypoxanthine	137.04	162.48	±	3.28	0.4	.015
Adenosine 5'‐diphosphate (ADP)	426.02	439.47	−	2.35	0.4	.001
L‐glutamine	145.06	347.84	−	1.65	0.21	.002
L‐glutamate	148.06	372.63	±	2.85	0.39	<.001
Deoxyguanosine triphosphate (dGTP)	505.99	463.13	−	2.07	0.51	.009
Uridine diphosphate glucose (UDP‐D‐glucose)	565.05	421.03	−	1.96	0.46	.001
Uridine 5'‐monophosphate (UMP)	323.03	417.94	−	1.47	0.34	<.001
Uridine	243.06	158.52	−	2.82	0.3	.004
Uracil	113.03	86.21	+	1.42	0.36	.013
Uridine 5'‐diphosphate (UDP)	402.99	452.34	±	2.18	0.32	.001
Uridine 5'‐triphosphate (UTP)	482.96	472.04	−	1.24	0.48	.004
Glycerophosphocholine	258.11	363.48	+	8.08	2.38	.002
sn‐Glycerol 3‐phosphoethanolamine	215.06	143.17	+	3.47	0.02	.001
Choline	104.11	363.48	+	1.38	1.92	.004
Phosphorylcholine	184.07	455.46	±	7.49	0.55	<.001
Glutathione disulphide	611.15	470.75	±	5.09	0.24	<.001
Glutathione	308.09	377.21	±	5.03	0.13	.009
L‐pyroglutamic acid	128.03	373.19	±	2.31	0.33	<.001
Nicotinamide adenine dinucleotide (NAD)	662.1	412.11	±	2.02	0.5	.001
Nicotinamide	123.05	65.3	+	1.93	0.63	.005
1‐Methylnicotinamide	137.07	278.4	+	3.1	0.37	.034
S‐methyl‐5'‐thioadenosine	296.08	48.84	±	2.26	0.35	<.001
1‐Aminocyclopropanecarboxylic acid	84.04	372.78	+	1.75	0.41	<.001
N‐acetyl‐L‐aspartic acid	174.04	369.4	−	3.44	0.23	<.001
Succinate	117.02	363.98	−	1.41	0.36	<.001
Phosphocreatine	212.04	416.63	+	1.65	0.21	<.001
Taurine	124.01	275.4	±	2.82	0.26	.001
Betaine	118.08	256.28	+	1.82	0.47	.011
Ketoisocaproic acid	129.05	41.54	−	6.87	3.57	.031
L‐valine	118.08	281.97	+	2.19	1.47	.03
cis‐9‐Palmitoleic acid	253.22	42.94	−	1.18	0.71	.01
Myristic acid	227.2	43.84	−	1.35	0.69	.014
Sphingomyelin (d18:1/18:0)	794.6	44.6	+	1.16	1.56	.048
Uridine 5'‐diphosphoglucuronic acid (UDP‐D‐glucuronate)	579.03	453.41	−	1.43	0.6	.002
Arachidonic acid (peroxide free)	303.23	41.45	−	7.3	3.13	.001
L‐malic acid	133.01	382.85	−	1.59	0.32	.007
Linoleic acid	279.23	42.1	−	4.03	1.61	.014
1‐Deoxy‐D‐xylulose 5‐phosphate	273.04	385.04	−	2.39	0.15	.024
Adenosine 3',5'‐diphosphate (PAP)	428.04	438.22	+	2.19	0.42	.001
1‐Oleoyl‐sn‐glycero‐3‐phosphocholine	522.36	138.27	+	5.52	0.26	<.001
N‐acetylglutamine	249.11	392.42	+	1.64	0.06	<.001
1‐Myristoyl‐sn‐glycero‐3‐phosphocholine	468.31	152.33	+	1.23	0.22	.001
1‐Palmitoyl‐sn‐glycero‐3‐phosphocholine	496.34	145.67	+	3.9	0.45	.001
Cyclic adenosine diphosphate ribose	540.05	412.11	−	1.37	0.51	.001
11(Z),14(Z)‐Eicosadienoic Acid	307.26	41.1	−	2.48	1.37	.001
7Z, 10Z, 13Z, 16Z, 19Z‐Docosapentaenoic acid	329.25	40.45	−	3.06	1.41	.002
Penicillin G	333.09	44.97	−	2.11	0.04	.003
1‐Stearoyl‐2‐hydroxy‐sn‐glycero‐3‐phosphocholine	524.37	135.57	+	1.96	0.43	<.001
1‐Stearoyl‐sn‐glycerol 3‐phosphocholine	568.34	131.65	+	1.75	0.13	<.001
1,2‐dioleoyl‐sn‐glycero‐3‐phosphatidylcholine	768.59	129.12	+	1.61	2.38	.014
1‐Palmitoyl‐2‐oleoyl‐phosphatidylglycerol	747.52	42.79	−	8.67	7.69	<.001
Ile‐Ala	203.14	41.63	+	1.5	0.27	.032
Myristoleic acid	225.18	43.57	−	1.35	0.43	<.001
Pentadecanoic acid	241.21	43.14	−	1.53	0.59	<.001
4‐Nonylphenol	219.17	32.12	−	2.2	3.03	<.001
Docosatrienoic acid	333.28	40.11	−	1.94	1.85	<.001
Adrenic acid	331.26	40.71	−	2.09	1.92	<.001
Cyclohexylamine	160.13	361.87	+	1.17	0.4	.001
DL‐lactate	89.02	222.39	−	3.46	0.5	.011

Abbreviations: FC, fold change; m/z, mass‐to‐charge ratio; Rt, retention time; VIP, variable importance in the projection.

### 3.8 Altered metabolic pathways in HJC0152‐treated NSCLC cell line A549

Pathway analysis using the Kyoto Encyclopedia of Genes and Genomes (KEGG) database was performed for the metabolites whose accumulation was increased in HJC0152‐treated A549 cells. The KEGG enrichment analysis showed that the metabolites that were significantly changed after HJC0152 treatment were highly associated with purine metabolism, ATP‐binding cassette (ABC) transport, pyrimidine metabolism and 17 other signalling pathways, as shown in Figure 8C. The major perturbed pathways are described in detail below.

#### 3.8.1 Purine metabolism

Purines and their derivatives are widely involved in biological processes, including host‐tumour interactions.^35^, ^36^ In the NSCLC A549 cell line, the metabolic pathway most affected by HJC0152 was purine metabolism, with significantly decreased levels of all identified intermediates, including adenosine monophosphate, adenosine, adenine, inosine, hypoxanthine, adenosine 5'‐diphosphate and L‐glutamine (Table 1), suggesting that HJC0152 downregulates purine metabolism.

#### 3.8.2 ATP‐binding cassette transporters

ATP‐binding cassette transporters are a large class of transmembrane proteins involved in substance transport^37^ and use ATP hydrolysis to transport diverse substrates (eg, amino acids, lipids, lipopolysaccharides, inorganic ions, polypeptides, carbohydrates and various drugs) across cell and organelle membranes, thus in association with many diseases and cancers.^38^ In HJC0152‐treated A549 cells, 8 altered metabolites were involved in ABC transporter pathways (Table 1). Among them, l‐glutamate and glutathione, which are involved in the glutathione metabolism pathway, were significantly reduced. The lower levels of glutathione found in HJC0152‐treated A549 cells suggest that HJC0152 decreases the cellular capacity to scavenge free radicals, resulting in enhanced ROS generation. These results suggest that the metabolic changes in response to HJC0152 are highly associated with ROS levels in A549 cells.

#### 3.8.3 Pyrimidine metabolism

Pyrimidine synthesis is important for DNA replication in tumour cells.^39^ The abundance of several metabolites involved in pyrimidine metabolism was decreased in A549 cells after HJC0152 exposure, including uridine diphosphate glucose, uridine 5'‐monophosphate, uridine, uracil, uridine 5'‐diphosphate and uridine 5'‐triphosphate (Table 1). These results demonstrate that HJC0152 causes perturbation of pyrimidine metabolism in A549 cells.

## 4 DISCUSSION

Based on the results obtained from this study, we concluded that HJC0152 exerts its anti‐cancer effect on human NSCLC at least partially via regulating STAT3 signalling and metabolic processes regulated by STAT3. We speculate that inhibition of STAT3 activation and reprogramming of STAT3‐regulated metabolic pathways are the predominant mechanisms associated with the in vitro and in vivo effect and efficacy of HJC0152 against NSCLC. Further analysis of HJC0152‐associated off‐target mechanisms is currently underway.

STAT3 mediates the signal transduction of many cytokines and growth factors to participate in cell growth, differentiation, proliferation, apoptosis and other molecular events important in carcinogenesis.^40^ Under normal circumstances, the activation of intracellular STAT3 signalling, which is controlled by a variety of negative regulatory factors, is rapid but transient. Recent studies have shown that STAT3 is abnormally and continuously activated in leukaemia, lung cancer, liver cancer, breast cancer and prostate cancer, and that constitutive activation of the STAT3 signalling pathway promotes malignant proliferation and invasion of cells, leading to tumorigenesis and tumour progression.^41^

On this basis, we speculated that HJC0152, a STAT3 inhibitor, might be a promising therapeutic agent for treating lung cancer, including NSCLC. To test our hypothesis, we investigated the anti‐cancer effect of HJC0152 against NSCLC. We first measured the effect of HJC0152 on cell growth of NSCLC cells. Our results suggested that HJC0152 treatment significantly inhibits NSCLC proliferation in dose‐ and time‐dependent manners. Next, we validated that expression levels of STAT3 were negatively correlated with progression‐free survival time in patients with lung adenocarcinoma. Moreover, STAT3 was more highly expressed in lung adenocarcinoma tissues than in corresponding normal tissue. Furthermore, we found that the inhibitory effect of HJC0152 on phosphorylation of STAT3 at Tyr705 was similar to that of STAT3 siRNA and that the effect of HJC0152 on NSCLC cells was dose dependent. These results demonstrated a key role for STAT3 in lung cancer, particularly in NSCLC, rendering STAT3 a valuable target for lung cancer treatment.

ROS generation plays a vital role in promoting apoptosis; this mechanism is frequently employed by various anti‐cancer agents to scavenge cancer cells.^17^, ^42^ We found that HJC0152 promotes intracellular ROS production and induces apoptosis in NSCLC cell lines A549 and H460 in a dose‐dependent manner. Double‐strand breaks are considered to be the most cytotoxic molecular events to cancer cells,^43^ and γ‐H2AX is a biomarker of these breaks. We used immunofluorescence staining to examine the formation of γ‐H2AX foci in NSCLC cells. HJC0152 treatment increased the formation of γ‐H2AX foci, consistent with the results of the immunofluorescence assay. These findings were further supported by the observation that the protein level of γ‐H2AX decreased in a dose‐dependent manner in HJC0152‐treated NSCLC cells, providing further evidence that HJC0152 induces DNA damage to suppress cancer cell proliferation and tumour growth and progression. It has been shown that activated STAT3 protects β‐cells from DNA damage in partial pancreatic duct ligation, the level of DNA damage in β‐cells increased after deletion of STAT3.^44^ Consistently, another study reported that HT29 cells treated with AG490, a specific inhibitor of STAT3 phosphorylation and activity, had abolished STAT3 phosphorylation and accumulation of γH2AX.^13^ In line with this, our results showed that decreased phosphorylation of STAT3 in NSCLC cells largely accelerated DNA damage, as monitored by γH2AX formation.

HJC0152 also inhibited the migration and invasion ability of A549 and H460 cells in a dose‐dependent manner. EMT is widely thought to be one of the most important mechanisms in cancer metastasis 32; thus, we measured levels of E‐cadherin, an epithelial phenotype marker, N‐cadherin and vimentin, markers of the acquired mesenchymal phenotype, and Twist, a nuclear transcription factor, in HJC0152‐treated NSCLC cells.^45^, ^46^ Our results demonstrated that HJC0152 treatment interrupted the EMT process during cancer invasion, thus providing a rationale for treating invasive, progressive and metastatic cancers with HJC0152.

To more comprehensively understand the effects of HJC0152 against NSCLC, we further performed xenograft tumour‐growth experiments in nude mice that had been subcutaneously injected with A549 cells and treated with vehicle or HJC0152. Our results showed that HJC0152 significantly reduced the weight of tumours in treated mice compared with those in untreated control mice, which confirmed the benefit of HJC0152 therapy in this xenograft tumour model. Moreover, HJC0152 reduced expression of p‐STAT3 (Tyr705), Ki67 and cleaved caspase‐3 in tumours. Collectively, these results suggest that HJC0152 is effective against NSCLC in vitro and in vivo.

The most exciting discovery of this study is that the metabolism of NSCLC cells was altered upon HJC0152 treatment. Accumulating evidence suggests that cancer is a metabolism‐related disease. Reprogramming of energy metabolism is a sign of cancer development and progression. Cancer cells may gain a growth advantage or escape apoptosis and other forms of cell death through alterations in their metabolic regulation.^7^ Given that STAT3 is a key regulator of cellular metabolism, we employed untargeted metabolomic analysis to study the effect of inhibiting it with HJC0152 on metabolites in A549 NSCLC cells. We found that HJC0152 caused significant alterations of the abundance of several metabolites, suggesting that HJC0152 can target metabolic processes. With metabolic profiling and further data mining, we found 47 metabolites in the negative mode and 38 metabolites in positive mode that were enriched in HJC0152‐treated cells. More specifically, we uncovered marked changes in the levels of several important metabolites, including glutamine, and in metabolic pathways including purine metabolism, ABC transporters and pyrimidine metabolism.

Recent reports have shown that glutamine, which is important for the synthesis of glutathione,^47^, ^48^ is an abundant and versatile nutrient that participates in the proliferation of cancer cells and their metabolism. Glutathione is riched in antioxidants in cancer cells and is important for cellular redox homeostasis and cancer cell survival in response to oxidative stress.^49^ Our results showed that HJC0152 treatment reduces the abundance of glutamine and glutathione among the metabolites in A549 cells. HJC0152 also decreases the antioxidant capacity of A549 cells and increases ROS levels. When ROS is accumulated to a level higher than the antioxidant capacity of cells, oxidative stress occurs, leading to apoptosis.^17^ Our results also showed that HJC0152 induces apoptosis in A549 NSCLC cells. Collectively, these observations demonstrate that the metabolic changes induced by HJC0152 cause apoptosis by promoting ROS generation in A549 cells. These findings are consistent with those of our recent report on metastatic breast cancer cells.^17^, ^20^ Additionally, we found a markedly high level of sphingomyelin (d18:1/18:0) in HJC0152‐treated A549 cells, strongly suggesting that HJC0152 also acts by targeting the key role of sphingomyelin in maintaining the integrity of the cell membrane for the protection of cells from apoptosis.

The findings of the current study support the notion that the anti‐cancer effect of HJC0152 against NSCLC may be primarily mediated by STAT3 signalling, a key regulator of cellular metabolism and ROS production. It has recently been shown that glutamine per se activates STAT3, which promotes cancer cell proliferation, and that STAT3 activation is independent of glutamine metabolism.^50^ This is not inconsistent with our results, which show that HJC0152 regulates not only STAT3, but also metabolites including glutamine in NSCLC cells.

With its efficacy against multiple cancer types, HJC0152 has the promise to be developed as a targeted therapy for single use or in combination with standard‐of‐care therapies. This study provides a solid rationale for the further investigation of HJC0152 as a treatment for lung cancer, particularly NSCLC. In addition, further study of how HJC0152 regulates explicit metabolite pathways is currently ongoing.

## CONFLICT OF INTEREST

The authors declare that they have no potential conflicts of interest.

## AUTHOR CONTRIBUTIONS

LL and QS conceived the study and generated hypotheses. LL designed and performed the experiments, and analysed the data with the help from HL, XW and JR provided assistance for metabonomic analysis. JZ provided HJC0152. LL, QS and SJF wrote the manuscript. All authors critically reviewed the content and approved the final version for publication.

## Data Availability

The data that support the findings of this study are available from the corresponding author upon reasonable request.
