# Supplementary figures and images for: HJC0152 suppresses human non–small‐cell lung cancer by inhibiting STAT3 and modulating metabolism
Source: Cell Prolif. 2020 Feb 5;53(3):e12777. doi: 10.1111/cpr.12777 (PMC7106968; doi:10.1111/cpr.12777)

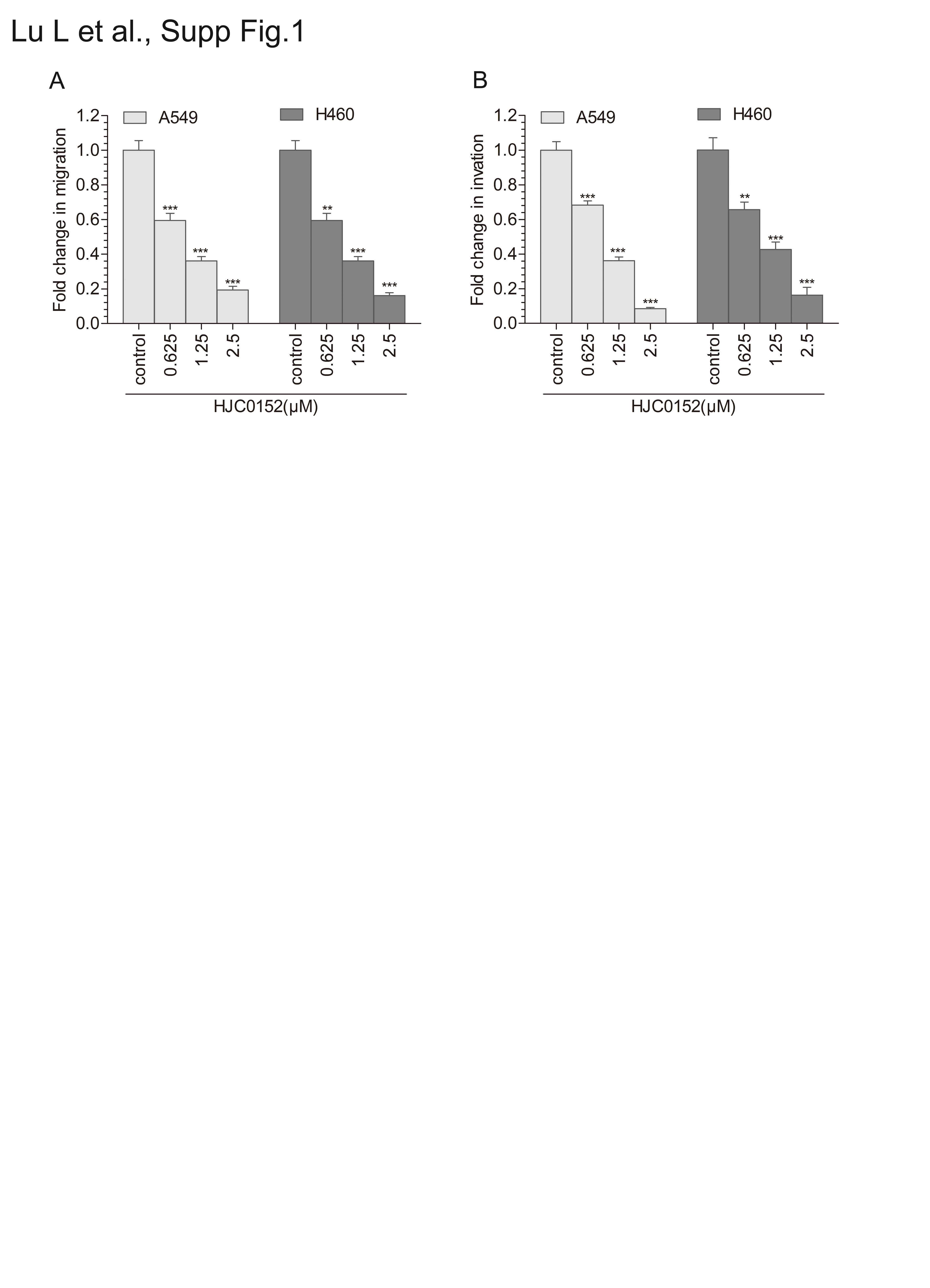

Supplement: Supplementary file 1 [file CPR-53-e12777-s001.jpg]

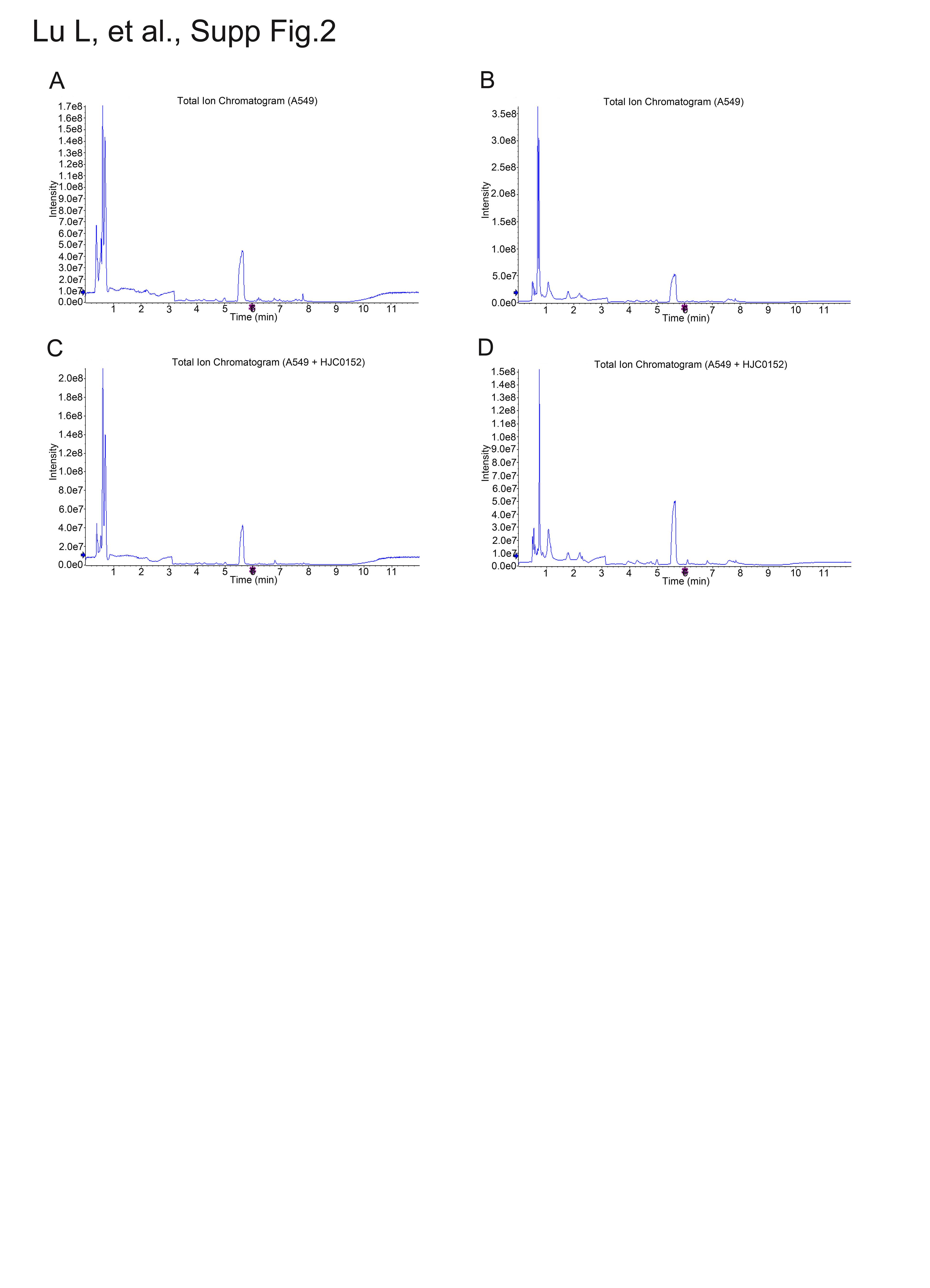

Supplement: Supplementary file 2 [file CPR-53-e12777-s002.jpg]
