# Supplementary material for: HJC0152 suppresses human non–small‐cell lung cancer by inhibiting STAT3 and modulating metabolism
Source: Cell Prolif. 2020 Feb 5;53(3):e12777. doi: 10.1111/cpr.12777 (PMC7106968; doi:10.1111/cpr.12777)
Supplement: Supplementary file 3 [file CPR-53-e12777-s003.docx]

**Supporting Information**

1. **Experimental Instruments and Softwares for the analysis of metabolomics samples by UHPLC-QTOF/MS**

| Instrument | UHPLC (1290 Infinity LC, Agilent Technologies) coupled to a quadrupole time-of-flight (AB Sciex TripleTOF 6600) | | |
| --- | --- | --- | --- |
| Analytical Column | HIIC: 2.1 mm × 100 mm ACQUIY UPLC BEH 1.7 µm column (waters, Ireland) | | |
|  | T3: 2.1 mm × 100 mm ACQUIY UPLC HSS T3 1.8 µm column (waters, Ireland) | | |
| Column Temperature | 25°C | | |
| Mobile Phases  (ESI positive mode) | A: water with 0.1% formic acid  B: acetonitrile with 0.1% formic acid | | |
| Mobile Phases  (ESI negative mode) | A: 0.5 mM ammonium fluoride in water  B: acetonitrile | | |
| Softwares： | SIMCA-P 14.1（Umetrics，Umea，Sweden）  XCMS | | |
| Gradient Profile | Time (min) | Percentage B (%) | Flow Rate (mL/min) |
|  | 0.0 | 1 | 0.30 |
|  | 1.5 | 1 | 0.30 |
|  | 13.0 | 99 | 0.30 |
|  | 16.5 | 99 | 0.30 |
|  | 16.6 | 1 | 0.30 |
|  | 20.0 | 1 | 0.30 |
| Injection Volume | 2 μL | | |

1. **MS/MS Analysis**

The ESI source conditions were set as follows: Ion Source Gas1 (Gas1) as 60, Ion Source Gas2 (Gas2) as 60, curtain gas (CUR) as 30, source temperature: 600℃, IonSpray Voltage Floating (ISVF) ± 5500 V. In MS only acquisition, the instrument was set to acquire over the m/z range 60-1000 Da, and the accumulation time for TOF MS scan was set at 0.20 s/spectra. In auto MS/MS acquisition, the instrument was set to acquire over the m/z range 25-1000 Da, and the accumulation time for product ion scan was set at 0.05 s/spectra. The product ion scan is acquired using information dependent acquisition (IDA) with high sensitivity mode selected. The parameters were set as follows: the collision energy (CE) was fixed at 35 V with ± 15 eV; declustering potential (DP), 60 V (+) and −60 V (−); exclude isotopes within 4 Da, candidate ions to monitor per cycle: 10.

**3. Bioinformatic Analysis**

**3.1 KEGG Pathway Annotation**

The metabolites were blasted against the online Kyoto Encyclopedia of Genes and Genomes (KEGG) database (http://geneontology.org/) to retrieve their COs and were subsequently mapped to pathways in KEGG11. The corresponding KEGG pathways were extracted.

**3.2 Functional Enrichment analysis**

To further explore the impact of differentially expressed metabolites, enrichment analysis was performed. KEGG pathway enrichment analyses were applied based on the Fisher’ exact test, considering the whole metabolites of each pathway as background dataset. And only pathways with p-values under a threshold of 0.05 were considered as significant.
